# Lysine-Triggered Polymeric Hydrogels with Self-Adhesion, Stretchability, and Supportive Properties

**DOI:** 10.3390/polym16101388

**Published:** 2024-05-13

**Authors:** Chieh-Yun Juan, You-Sheng Zhang, Jen-Kun Cheng, Yu-Hsu Chen, Hsin-Chieh Lin, Mei-Yu Yeh

**Affiliations:** 1 Department of Chemistry, Chung Yuan Christian University, No. 200, Zhongbei Rd., Zhongli Dist., Taoyuan City 320314, Taiwan; jieyunr@gmail.com (C.-Y.J.); brian20010418@gmail.com (Y.-S.Z.); 2 Department of Medical Research, MacKay Memorial Hospital, Taipei 10449, Taiwan; jkcheng@usa.net; 3 Department of Anesthesiology, MacKay Memorial Hospital, Taipei 10449, Taiwan; 4 Department of Medicine, MacKay Medical College, New Taipei City 25245, Taiwan; 5 Department of Orthopedic Surgery, Taoyuan General Hospital, Ministry of Health and Welfare, Taoyuan 330215, Taiwan; 6 Department of Biology and Anatomy, National Defense Medical Center, Taipei 114201, Taiwan; 7 Department of Materials Science and Engineering, National Yang Ming Chiao Tung University, Hsinchu 300093, Taiwan; 8 Center for Intelligent Drug Systems and Smart Bio-Devices (IDS2B), National Yang Ming Chiao Tung University, Hsinchu 30068, Taiwan

**Keywords:** lysine, adhesive, stretchable, hydrogel, fatigue resistance

## Abstract

Hydrogels, recognized for their flexibility and diverse characteristics, are extensively used in medical fields such as wearable sensors and soft robotics. However, many hydrogel sensors derived from biomaterials lack mechanical strength and fatigue resistance, emphasizing the necessity for enhanced formulations. In this work, we utilized acrylamide and polyacrylamide as the primary polymer network, incorporated chemically modified poly(ethylene glycol) (DF-PEG) as a physical crosslinker, and introduced varying amounts of methacrylated lysine (LysMA) to prepare a series of hydrogels. This formulation was labeled as poly(acrylamide)-DF-PEG-LysMA, abbreviated as pADLx, with x denoting the weight/volume percentage of LysMA. We observed that when the hydrogel contained 2.5% *w*/*v* LysMA (pADL2.5), compared to hydrogels without LysMA (pADL0), its stress increased by 642 ± 76%, strain increased by 1790 ± 95%, and toughness increased by 2037 ± 320%. Our speculation regarding the enhanced mechanical performance of the pADL2.5 hydrogel revolves around the synergistic effects arising from the co-polymerization of LysMA with acrylamide and the formation of multiple intermolecular hydrogen bonds within the network structures. Moreover, the acid, amine, and amide groups present in the LysMA molecules have proven to be instrumental contributors to the self-adhesion capability of the hydrogel. The validation of the pADL2.5 hydrogel’s exceptional mechanical properties through rigorous tensile tests further underscores its suitability for use in strain sensors. The outstanding stretchability, adhesive strength, and fatigue resistance demonstrated by this hydrogel affirm its potential as a key component in the development of robust and reliable strain sensors that fulfill practical requirements.

## 1. Introduction

Hydrogels, a unique class of flexible materials, have emerged as a prominent subject of research in recent years. They possess many distinctive properties, such as highly tunable physical and chemical characteristics, as well as biocompatibility, which allows them to meet the needs of various applications and hold immense potential. For example, in the field of wearable strain sensors and electronic skins, the softness and stretchability of hydrogels make them ideal sensing materials. Hydrogels can conform closely to the skin or surface of organs and can detect external strains by monitoring the deformation of the gel, thus offering potential applications in health monitoring, sports training, and medical diagnostics [1,2,3,4,5,6,7,8,9,10]. In tissue engineering, hydrogels can serve as scaffolds or carriers to support cell growth and differentiation. Their three-dimensional network structure can mimic the microenvironment of natural tissues, promoting the physiological functions of cells [7,8]. In terms of bioadhesives, hydrogels can enhance their adhesive properties through surface modifications. They are also compatible with biological tissues, reducing damage to tissues. This makes hydrogels potentially valuable in surgical procedures and wound healing [9,10].

Mechanical performance is crucial in flexible strain sensors, including factors such as stretchability, supportability, and adhesiveness [11,12,13]. However, most hydrogel sensors prepared from biomaterials often exhibit subpar performance in these aspects. This may be due to the hydrogel’s network structure not being able to effectively recover to its original state during stretching or twisting or failing to provide sufficient support when adhered to different surfaces. Additionally, since the covalent bonds formed in the hydrogel network are typically irreversible, this limits the hydrogel’s fatigue resistance in long-term applications [14,15,16]. Once the hydrogel is subjected to multiple stretches or twists, its network may gradually fatigue, leading to a decrease in its performance and lifespan. Therefore, developing a hydrogel with good deformability and fatigue resistance is crucial for the application of strain sensors. Such a hydrogel should be able to recover to its original state after multiple deformations and provide sufficient support when adhered to a surface, ensuring stable performance of the sensor over long-term use. Improving the mechanical performance of hydrogels is a challenging yet highly worthwhile research direction. This goal may be achieved by designing the internal crosslinking mechanisms (i.e., non-covalent bonding forces) and chemical composition of the hydrogel [17], for example, by utilizing hydrophobic interactions [18,19], intermolecular hydrogen bonds [20,21,22,23], ion interactions [24,25,26], metal coordination chemistry [27,28,29,30], or adjusting the crosslinking density to modify the properties of the hydrogel [31,32]. This will help enhance the application value of hydrogels in fields such as flexible strain sensors and promote the development and application of related technologies.

Lysine is an essential amino acid for the human body, known for its excellent water solubility and biocompatibility. It plays crucial roles in various human mechanisms, including aiding in the absorption of calcium, iron, and zinc, promoting collagen growth, enhancing athletic performance and muscle growth, assisting in enzyme and antibody production, and strengthening the immune system [33,34,35]. In addition, the free amine and carboxyl groups on the lysine molecule can serve as hydrogen bond donors and acceptors, allowing it to easily form hydrogen bonds with hydrophilic molecules, thereby providing additional mechanical strength and adhesion [36]. In recent years, lysine-based hydrogels have been widely studied. For example, Chi and Xu developed a biomimetic mussel-inspired ε-poly-_L_-lysine hydrogel, demonstrating its excellent tissue-anchor and anti-infection abilities [33]. Yao and Yang et al. designed a deoxyribonucleic acid/poly-(_L_-lysine) hydrogel, which extended the 3D cell culture from generally 1 day to longer than 15 days [35].

On the other hand, hydrogels solely composed of acrylamide (AAM) exhibit strong water absorption capacity but poor mechanical performance [37]. To address this issue, in our study, we chemically modified lysine to synthesize methacrylated lysine (LysMA). The goal was to enable covalent crosslinking copolymers with AAM, aiming to densify the crosslinking network and enhance mechanical performance [38]. Moreover, we introduced dialdehyde-functionalized poly(ethylene glycol) (DF-PEG) to provide additional physical crosslinking in the hydrogel, such as π-π interactions, intermolecular hydrogen bonds, and hydrophobic interactions, enhancing its mechanical properties [39]. In addition, recent studies have utilized polyacrylamide-PEG-based hydrogels for protein adsorption, drug release carriers, etc., demonstrating their feasibility in biomedical applications [40,41]. Our objective was to develop hydrogels with excellent deformability and fatigue resistance by optimizing the chemical composition. We found that changing the amount of LysMA significantly improved the hydrogel’s performance. Compared to hydrogels without LysMA (pADL0), the optimized pADL2.5 hydrogel exhibited a 642 ± 76% increase in stress, a 1790 ± 95% increase in strain, and a 2037 ± 320% increase in toughness. Furthermore, in the tensile test, pADL2.5 could be stretched up to 2600% of its original length and could extend to 1000% of its original length after knotting. Finally, in fatigue resistance experiments, good recovery properties were observed at 100%, 200%, 500%, or 800% strain, demonstrating pADL2.5’s excellent stability and fatigue resistance.

## 2. Materials and Methods

### 2.1. Materials

Acrylamide (AAM) and polyacrylamide (PAAM, average MW 10000) were obtained from Acros Organics (Geel, Antwerp, Belgium), while polyethylene glycol (PEG, average MW 2000), *N-N’*-Dicyclohexylcarbodiimide (DCC) were sourced from Alfa Aesar (Ward Hill, MA, USA). Methacrylic anhydride, 4-formylbenzoic acid, and potassium persulfate (PPS) were acquired from Sigma-Aldrich (St. Louis, MO, USA). The compound N-Boc-_L_-Lysine was purchased from Carbosynth Ltd. (Compton, UK).; 4-dimethylaminopyridine (DMAP) was obtained from Matrix Scientific (Elgin, SC, USA). Deionized water was utilized in all experiments.

### 2.2. Synthesis of DF-PEG and LysMA

The synthesis of DF-PEG was conducted according to reference [39]. PEG (3.00 g, 1.50 mmol) and 4-formylbenzoic acid (1.35 g, 8.99 mmol) were added to a two-neck flask along with DMAP (0.11 g, 0.90 mmol). The mixture was then dissolved in 30 mL of dichloromethane (DCM) under a nitrogen atmosphere and stirred until homogeneous. DCC (1.85 g, 8.97 mmol) was slowly added dropwise to the solution, and the reaction was allowed to proceed at room temperature for 24 hours. The precipitate in the reaction solution was filtered off to obtain a filtrate, which was precipitated several times with ice ether. The white solid obtained after vacuum filtration was then vacuum dried to yield the product (DF-PEG), with a yield of 56% (1.92 g). ^1^H NMR (400 MHz, CDCl_3_): δ 10.11 (s, 2H), 8.22 (d, *J*= 7.8 Hz, 4H), 7.96 (d, *J* = 7.8 Hz, 4H), 4.51 (t, *J* = 4.2 Hz, 4H), 3.85 (t, *J* = 4.2 Hz, 8H), and 3.64 (s, 241H).

The synthesis of LysMA followed the procedure outlined in reference [28]. N-Boc-_L_-Lys (0.50 g, 2.00 mmol) and NaHCO_3_ (0.34 g, 4.10 mmol) were dissolved in a mixture of tetrahydrofuran (THF) and deionized water (7.00 mL, *v*/*v* = 6/1). Methacrylic anhydride (3.95 mL) was dissolved in 7.5 mL of THF and added dropwise to the reaction solution at 0 °C over 15 min. After 24 h at room temperature, the THF was removed using a rotary evaporator, and the pH of the sample was adjusted to 2 with 0.1 M HCl aqueous solution. The sample was then extracted with DCM several times to extract the organic layer. The organic layer was washed several times with deionized water to remove impurities, yielding a yellowish crude product. Trifluoroacetic acid (15 mL) and DCM (15 mL) were added and stirred at room temperature for 2 h to remove the protective group. A mixture of n-hexane (15 mL) and ether (15 mL) was then added, and the mixture was stored in a refrigerator at −20 °C for 6 h. After removing the upper clear solution, a white viscous solid was obtained, which was dissolved in ethanol. Triethylamine was added to precipitate the product. After centrifugation, the upper clear solution was removed, and the solid was washed several times with ice-cold ethanol to yield a white powder with a yield of 39% (0.17 g). ^1^H NMR (400 MHz, D_2_O): δ 5.56 (s, 1H), 5.33 (s, 1H), 3.63 (t, *J* = 6.2 Hz, 1H), 3.17 (t, *J* = 6.2 Hz, 2H), 1.74–1.82 (m, 5H), 1.44–1.53 (m, 2H), 1.28–1.36 (m, 2H).

### 2.3. Preparation of Hydrogels

The pADL hydrogels were prepared through the radical polymerization of AAM, and LysMA in the presence of DF-PEG as a cross-linker and PPS as a radical initiator in deionized water. Based on the amount of LysMA, the hydrogels were named pADL0, pADL0.5, pADL1, pADL1.5, pADL2, pADL2.5, and pADL3. The specific compositions of the hydrogels are listed in Table S1 (see Supporting Information).

### 2.4. Characterizations and Mechanical Measurements

Nuclear magnetic resonance (NMR) spectra were acquired using a Bruker AVANCE II-400 MHz spectrometer, with D_2_O or CDCl_3_ as solvents. Tensile testing of the pADL hydrogels was conducted using a Gotech AI-3000-U tensile tester. For mechanical and adhesion testing, samples were cut into a square shape (20 mm × 20 mm) with a height of 2 mm. The tests were conducted at extension rates of 100 mm/min and 10 mm/min, respectively. Molecular interactions within the pADL hydrogels were evaluated using a Thermo Fisher Scientific Nicolet iS5 Fourier transform infrared (FT-IR) spectrometer. The samples were dissolved in D_2_O and examined using ZnSe (20 mm × 2 mm), and the spectra were obtained within the wavenumber range of 4000 to 500 cm^–1^ with a resolution of 1 cm^–1^. The morphologies of the hydrogels were examined by scanning electron microscopy (SEM) with a JSM-7600F model. To prepare the test samples, the hydrogels were subjected to freeze vacuum drying: they were rapidly frozen in liquid nitrogen and lyophilized in a freeze dryer system under vacuum conditions at −80 °C for at least 24 h to ensure complete water sublimation.

## 3. Results and Discussion

### 3.1. Design and Preparation of pADL Hydrogels

**Scheme 1:**
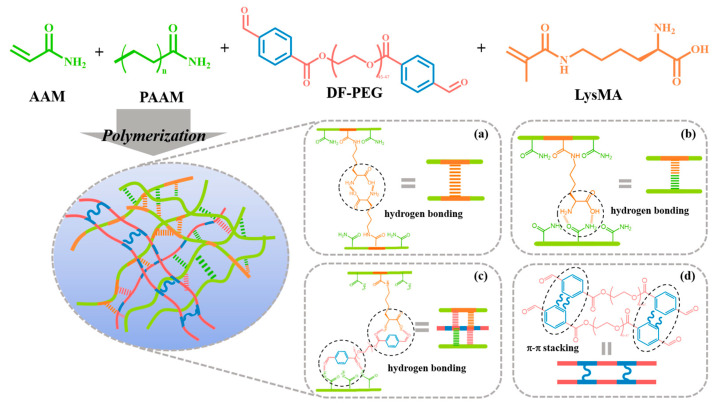
Schematic diagram of the pADL hydrogel, illustrating (**a**) intermolecular hydrogen bonding between LysMA and LysMA, (**b**) intermolecular hydrogen bonding between LysMA and PAAM, (**c**) intermolecular hydrogen bonding between LysMA and DF-PEG and/or PAAM and DF-PEG, and (**d**) π–π stacking between DF-PEG and DF-PEG.

**Figure 1:**
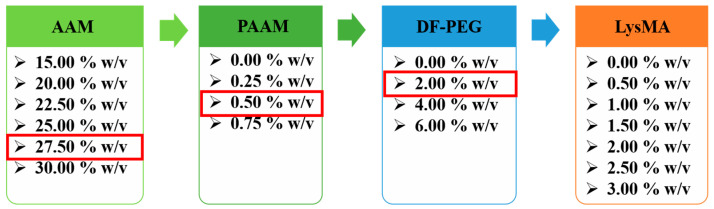
Flow chart for optimizing the formulation of pADLx hydrogels. (where x represents the amount of LysMA: pADL0 indicates 0.00% *w*/*v* LysMA, pADL0.5 indicates 0.50% *w*/*v* LysMA, pADL1 indicates 1.00% *w*/*v* LysMA, pADL1.5 indicates 1.50% *w*/*v* LysMA, pADL2 indicates 2.00% *w*/*v* LysMA, pADL2.5 indicates 2.50% *w*/*v* LysMA, and pADL3 indicates 3.00% *w*/*v* LysMA).

**Figure 2:**
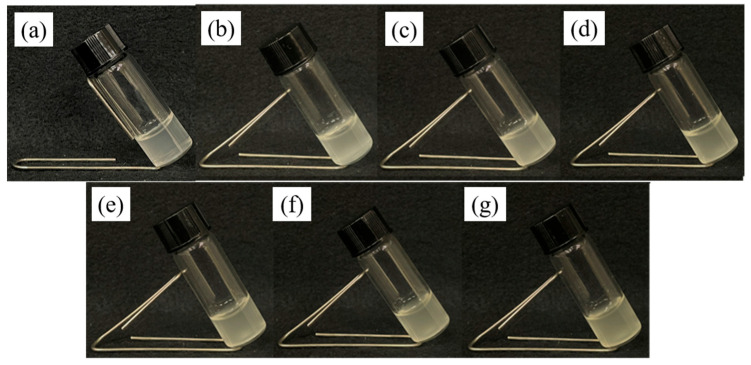
Optical images of pADLx hydrogels. (**a**) pADL0, (**b**) pADL0.5, (**c**) pADL1, (**d**) pADL1.5, (**e**) pADL2, (**f**) pADL2.5, and (**g**) pADL3.

In our prior investigation, we successfully created self-healing hydrogels; nevertheless, these hydrogels lacked adhesive properties [39]. Moreover, in this current material optimization process, our objective was to enhance the hydrogel’s resistance to fatigue in order to prolong its durability. In this study, alongside the utilization of AAM, PAAM, and DF-PEG, we introduced LysMA. As depicted in Scheme 1, we hypothesized that incorporating LysMA would increase the intermolecular hydrogen bonding forces, thereby bolstering the fatigue resistance of the targeted hydrogel. Additionally, the amine and acid groups present in LysMA hold promise for enhancing adhesion to various substrates. To further enhance the aforementioned properties of the hydrogel, we refined the formulation of the hydrogel (Figure 1). Firstly, we enhanced the hydrogels by adjusting the AAM content. As depicted in Figure S1 and Table S1, all hydrogels appeared translucent and uniform. However, it was observed that at 15.00% *w*/*v* AAM, the hydrogel flowed like glue when the bottle was tilted, indicating poor gelling. Although 20.00% *w*/*v* and 22.50% *w*/*v* AAM showed better formability, they failed to maintain their original shape and were very soft. On the other hand, hydrogels with 25.00% *w*/*v* and 27.50% *w*/*v* AAM were more intact compared to the previous ones, with 27.50% *w*/*v* AAM displaying greater elasticity and stretchability than 25.00% *w*/*v* AAM. Hydrogels with 30.00% *w*/*v* AAM exhibited the highest toughness among all, but their stretchability and adhesiveness were limited. Overall, we observed that as the AAM content increased, the elasticity and toughness of the hydrogel improved, which is beneficial for the subsequent mechanical properties to be explored. Therefore, we ultimately chose to continue optimizing the hydrogel with 27.50% *w*/*v* AAM, which exhibited better elasticity and stretchability. Next, we investigated the effect of adding PAAM on the overall mechanical properties of the hydrogel. Since PAAM is a pre-polymerized water-soluble polymer with a high molecular weight, its addition can enhance the toughness of the hydrogel, a result that was confirmed by our experimental findings. As revealed in Figure S2 and Table S1, the hydrogel’s formability exhibited better performance with increasing concentrations of PAAM, ranging from 0.00% *w*/*v* to 0.75% *w*/*v*, and could withstand greater strains, with the optimal results observed at 0.50% *w*/*v* and 0.75% *w*/*v* PAAM. Finally, we selected the hydrogel containing 0.50% *w*/*v* PAAM for subsequent studies. After confirming the amounts of AAM and PAAM, we proceeded to optimize the quantity of DF-PEG. We noticed that increasing the amount of DF-PEG resulted in the hydrogel becoming more opaque (Figure S3 and Table S1). Moreover, we found that once the concentration of DF-PEG exceeded 2.00% *w*/*v*, its tensile strength performance remained consistent. As a result, we decided to maintain the use of 2.00% *w*/*v* DF-PEG. Finally, we added LysMA to the above hydrogels (27.50% *w*/*v* AAM, 0.50% *w*/*v* PAAM, and 2.00% *w*/*v* DF-PEG) and studied its effect on their mechanical properties. We named this formulation poly(acrylamide)-DF-PEG-LysMA, abbreviated as pADLx, where x represents the weight percentage of LysMA. As shown in Figure 2 and Table S1, these seven hydrogels appeared translucent, indicating a uniform distribution facilitated by LysMA’s good solubility in water.

### 3.2. Mechanical Properties of pADL Hydrogels

**Figure 3:**
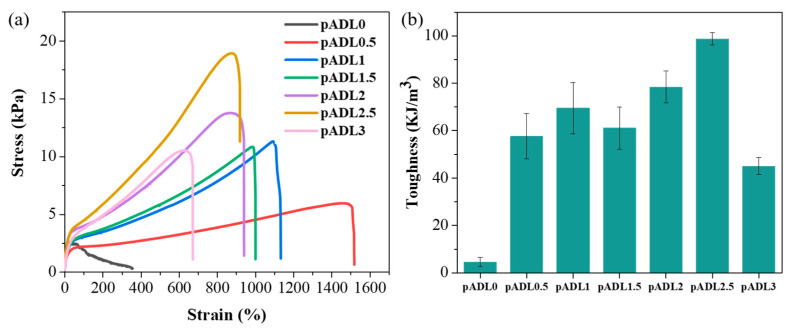
(**a**) Tensile stress–strain curves of pADLx hydrogels. (Black for pADL0, red for pADL0.5, blue for pADL1, green for pADL1.5, violet for pADL2, brown for pADL2.5, and pink for pADL3) (**b**) Toughness of pADL hydrogels (*n* = 3).

We conducted tensile tests on pADLx hydrogels using a tensile testing machine, with all tests performed at a rate of 100 mm/min (Figure 3 and Figure S4) [42,43]. As shown in Figure 3a and Table S2, hydrogels without LysMA (pADL0) exhibited extremely poor stretchability, breaking quickly during the stretching process. The addition of LysMA significantly improved the stretchability, with the maximum strain reaching 1521 ± 83% for pADL0.5, 30 times that of pADL0, indicating the beneficial effect of LysMA on the stretchability of the hydrogels. However, as the amount of LysMA increased, the strain at break gradually decreased. In terms of tensile stress, there was a gradual increase from pADL0.5 to pADL2.5 hydrogels. The stress at break for pADL2.5 increased to 18.9 ± 2.1 kPa, which was 642 ± 76% higher than that of pADL0, indicating an increase in strength due to the increased crosslinking density [44]. The pADL2.5 hydrogel was less prone to fracture, but the addition of 3.00% *w*/*v* LysMA resulted in a decrease in tensile stress. The decrease in tensile stress observed with the addition of 3.00% *w*/*v* LysMA to pADL0 may be attributed to the disruption of the crosslinking network. Excessive LysMA could potentially interfere with the crosslinking process, leading to a less cohesive network and reduced overall strength [44]. Thus, we found that the pADL2.5 hydrogel exhibited the best performance in terms of stretchability, as it could withstand the highest tensile stress while maintaining a moderate strain. The typical strain range of human skin is approximately 0 to 70% [45], indicating that the stretchability of pADLx hydrogels is advantageous for applications in devices such as electronic skins that need to withstand various degrees of human movement. Additionally, we calculated the slope of the stress–strain curve and the area under the curve to obtain the Young’s modulus [46] and toughness [47] values (Figure 3b, Figure S5 and Table S2). It was observed that the Young’s modulus showed a trend of increasing with increasing LysMA content before decreasing, and we found that both the Young’s modulus and toughness reached their maximum values for pADL2.5 hydrogels, at 8.7 ± 1.2 kPa and 98.8 ± 2.7 kJ/m^3^, respectively. Importantly, the toughness of pADL2.5 increased by 2037 ± 320% compared to pADL0.

### 3.3. Morphologies and Spectral Analysis of pADL Hydrogels

**Figure 4:**
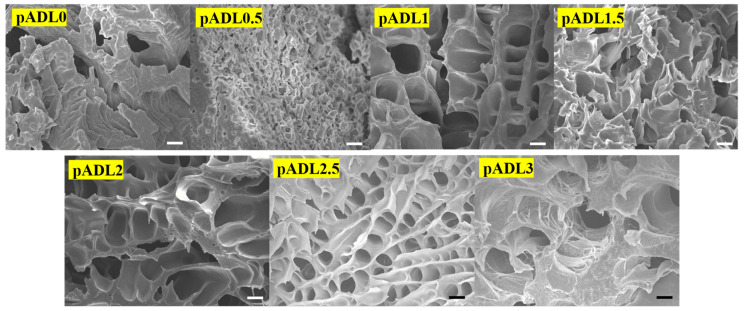
SEM images of pADLx hydrogels (Scale bar: 10 μm).

Subsequently, we examined the cross-sectional scanning electron microscope (SEM) images of the samples after freeze-drying (Figure 4). This information allows us to understand how the size and distribution of pores are controlled by the crosslinking density, revealing the relationship between morphology and crosslinking networks. Additionally, we delved deeper into the analysis by investigating the pore sizes of pADLx hydrogels in SEM images using ImageJ software (1.8.0_345). Following this, the data underwent rapid processing with Origin software (OriginPro 2016) to determine their accurate pore distribution (Figure S6) [48,49]. In Figure 4 and Figure S6, the cross-section of the pADL0 hydrogel appears as loosely stacked layers at the microscopic level, indicating weaker mechanical strength of the hydrogel. After the addition of LysMA, there is a significant change in the cross-sectional morphology, showing a porous structure. This is because the participation of LysMA increases the crosslinking density, demonstrating the structural stability of pADLx hydrogels. The pADL0.5 hydrogel exhibits smaller and more continuous pore structures compared to other ratios of hydrogels at the same magnification, maximizing strain in tensile tests but with minimal stress. As for pADL1.0 to pADL2.5 hydrogels, a trend can be observed. With an increasing LysMA ratio, the pore size becomes smaller and more densely packed, resulting in higher strength. In contrast, the cross-section of pADL3.0 shows a loose, large pore structure. These SEM image data are consistent with the results of the tensile tests, where the stress performance initially increases and then decreases with the amount of LysMA (Figure 3).

**Figure 5:**
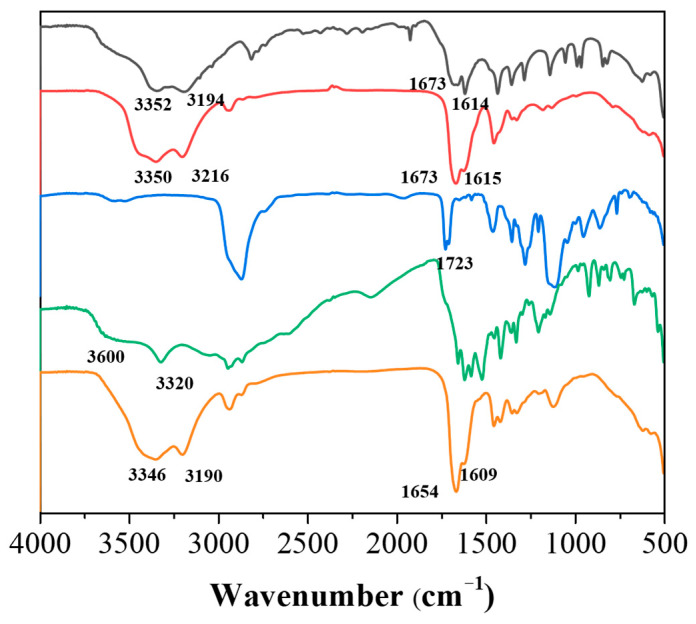
FT-IR spectra of AAM (black), PAAM (red), DF-PEG (blue), LysMA (green), and pADL2.5 (orange).

Since the pADL2.5 hydrogel exhibited the best mechanical properties, we further analyzed its composition by comparing the Fourier-transform infrared spectroscopy (FT-IR) spectra of each component with those of the pADL2.5 hydrogel. This comparison aimed to demonstrate the interactions between the molecules used and provide insights into the molecular structure and bonding within the hydrogel. As shown in Figure 5, the FT-IR spectrum of the pADL2.5 hydrogel exhibited two broad peaks at 3346 cm^−1^ and 3190 cm^−1^, as well as a signal at 1654 cm^−1^, corresponding to the N–H stretching and the C=O stretching vibration of AAM and PAAM, respectively [50]. Compared to the N–H and C=O signals of AAM and PAAM, the N–H and C=O signals of pADL2.5 shifted to lower wavenumbers, indicating the possible formation of hydrogen bonds in pADL2.5 [51,52,53]. Furthermore, in the spectrum of LysMA, characteristic peaks at 3600 cm^−1^, 3320 cm^−1^, and 1655–1450 cm^−1^ were observed, corresponding to O–H stretching, N–H stretching, C=O stretching, and C=C stretching, respectively. Apart from observing lower wavenumbers in pADL2.5 compared to LysMA, a more significant observation was the disappearance of the characteristic peak at 1615 cm^−1^ after the formation of the pADL2.5 hydrogel. We believe this is due to the copolymerization of LysMA with AAM, leading to the disappearance of the C=C originally present in LysMA [54].

### 3.4. Adhesive Capacities of pADL Hydrogels

**Figure 6:**
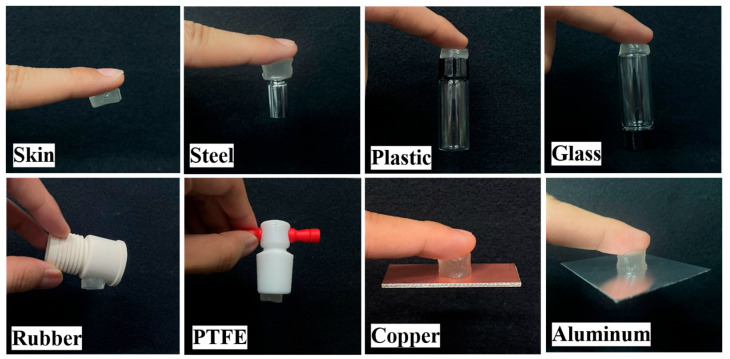
The adhesive properties of the pADL2.5 hydrogel were evaluated on various substrates.

**Figure 7:**
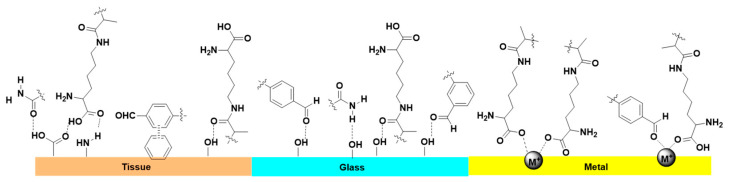
The proposed interface adhesion mechanism of the pADL2.5 hydrogel to various substrates.

Stable adhesion depends on the synergistic interaction between the surface structure of the material and the hydrogel, as well as the adhesive and cohesive forces within the hydrogel itself [55]. Typically, adhesion is more advantageous on rough surfaces, where the chemical interactions and mechanical interlocking between the hydrogel and the surface play a significant role [56]. However, adhesion on smoother surfaces often presents greater challenges. Figure 6 shows the results of our tests using pADL2.5 to adhere to various materials. It can be observed that pADL2.5 not only exhibits good adhesion on rough surfaces but also performs well on smoother materials such as aluminum and copper. Additionally, the hydrogel can adhere firmly to the skin without being affected by hand movements, which is beneficial for biomedical applications. To provide a more objective assessment of this series of hydrogels, we adhered the hydrogel between two aluminum metal pieces with a fixed area of 2 cm × 2 cm and performed adhesion tests using a tensile testing machine at a speed of 10 mm/min. As depicted in Figure S7, the lap-shear strength test of the pADL2.5 hydrogel on various substrates shows measured values ranging from 17.8 to 24.4 kPa. In addition, the proposed adhesion mechanism between pADL2.5 and substrates is illustrated in Figure 7 [57]. The adhesion strength of pADL2.5 was found to be 17.8 ± 2.9 kPa on skin (Figure S7), demonstrating its ability to maintain strong adhesion even under mechanical stress, which is promising for applications requiring durable adhesion, such as in biomedical devices and skin adhesives.

### 3.5. Stretchability and Supportiveness of pADL Hydrogels

**Figure 8:**
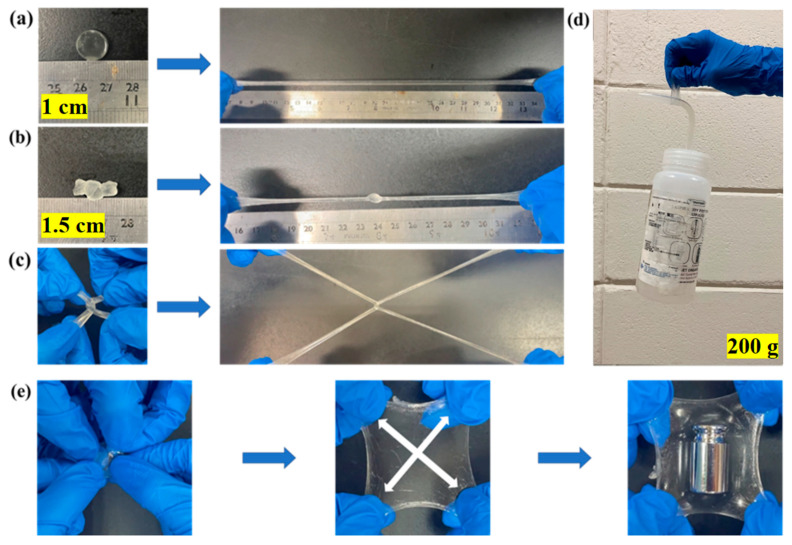
The pADL2.5 hydrogel was tested using the following methods: (**a**) stretching, (**b**) knotting and stretching, (**c**) crossing and stretching, (**d**) loading with a 200 g water bottle, and (**e**) spreading out the hydrogel and loading it with a weight.

**Figure 9:**
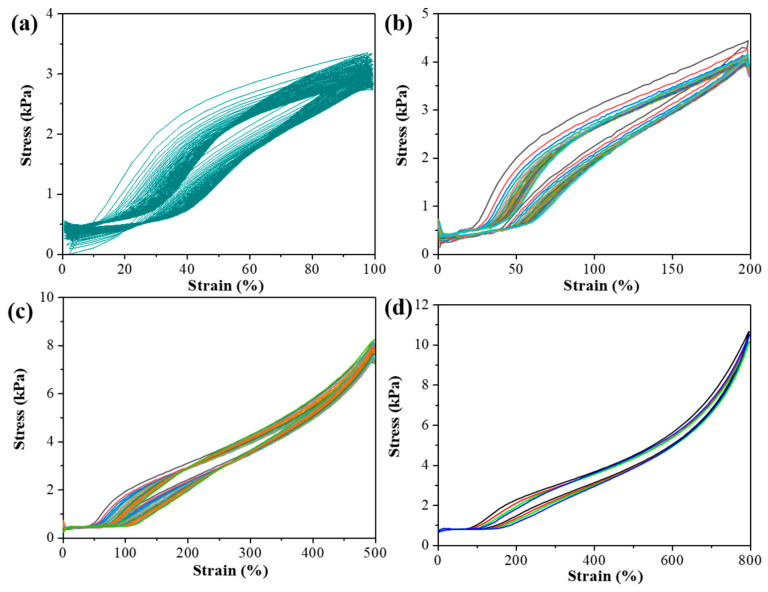
The stress–strain curves of the pADL2.5 hydrogel were obtained for loading-unloading cycles under varied strains: (**a**) 100% for 100 cycles, (**b**) 200% for 20 cycles, (**c**) 500% for 15 cycles, and (**d**) 800% for 5 cycles.

The experimental results above demonstrate the excellent mechanical properties of pADL2.5. We showcased its mechanical strength through various stretching methods. Figure 8a shows the longitudinal stretching of the hydrogel, which can be stretched up to 2600% of its original length. Additionally, the hydrogel can extend to 1000% of its original length after knotting (Figure 8b). Figure 8c depicts the cross-stretching of two hydrogel pieces, which did not break during the process, indicating outstanding tensile strength and toughness. Figure 8d,e shows load-bearing tests, where pADL2.5 can lift a water bottle weighing 200 g (Figure 8d). Interestingly, when the hydrogel is simultaneously stretched from four sides, it can withstand a load of at least 50 g of weight (Figure 8e and Video S1). To further investigate the fatigue resistance of pADL2.5, we conducted loading-unloading cycles using a tensile testing machine. Figure 9 shows the strain-stress curves from the cyclic test, highlighting a notable feature of pADL2.5: The hysteresis loop in the curve, indicating its ability to recover to a state close to the initial one after deformation. The area between the loading and unloading curves provides an indirect measure of the energy dissipated by the hydrogel during the process, which reflects its fatigue resistance [58]. In Figure S8, it can be observed that the energy dissipated by pADL2.5 during the first cycle of stretching is slightly higher than in subsequent cycles within the tested strain range. This suggests that pADL2.5 has good recovery properties, as the hydrogen bonds between molecules act as sacrificial bonds to dissipate energy and protect the polymer network from damage [59], demonstrating the good stability and high fatigue resistance of pADL2.5.

## 4. Conclusions

In summary, we successfully utilized AAM and PAAM as the primary polymer network, incorporated DF-PEG as a physical crosslinker, and introduced varying amounts of LysMA to prepare a series of hydrogels, namely pADL0, pADL0.5, pADL1, pADL1.5, pADL2, pADL2.5, and pADL3. We found that the inclusion of 2.5% *w*/*v* LysMA in the hydrogel (pADL2.5) significantly enhanced its mechanical properties compared to hydrogels without LysMA (pADL0), with stress increasing by 642 ± 76%, strain by 1790 ± 95%, and toughness by 2037 ± 320%. In the adhesive study, it was discovered that pADL2.5 demonstrates strong adhesion not only on rough surfaces but also on smoother materials like aluminum and copper. Additionally, in fatigue resistance tests, pADL2.5 showed good recovery properties at 100%, 200%, 500%, or 800% strain, indicating its excellent stability and fatigue resistance. These findings highlight pADL2.5’s potential for use in strain sensors, given its outstanding mechanical properties, including remarkable stretchability, adhesive strength, and fatigue resistance, making it a promising component for the development of robust and reliable strain sensors.

## Data Availability

Data are contained within the article.

## Supplementary Materials

The following supporting information can be downloaded at: https://www.mdpi.com/article/10.3390/polym16101388/s1, Table S1: Design of formulation; Figure S1: Optical images of hydrogels containing different % *w*/*v* of AAM; Figure S2: Optical images of hydrogels containing different % *w*/*v* of PAAM; Figure S3: Optical images of hydrogels containing different % *w*/*v* of DF-PEG; Figure S4: Comparison of tensile tests before and after stretching: (a) pADL0.5, (b) pADL1, (c) pADL1.5, (d) pADL2, (e) pADL2.5, and (f) pADL3. (Left: original length of 1 cm. Right: length after stretching); Figure S5: Young’s modulus of pADL hydrogels; Table S2 Mechanical properties of pADLx hydrogels; Figure S6: The porous diameter distributions of hydrogels of (a) pADL0, (b) pADL0.5, (c) pADL1, (d) pADL1.5, (e) pADL2, (f) pADL2.5, and (g) pADL3; Figure S7: Lap-shear strength test of the pADL2.5 hydrogel on various substrates (red for glass, blue for skin, and brown for aluminum); Figure S8: The dissipated energy of the pADL2.5 hydrogel; Figure S9: ^1^H NMR spectrum of DF-PEG in CDCl_3_; Figure S10: ^1^H NMR spectrum of LysMA in D_2_O; Video S1: Testing the stretchability and supportive properties of pADL2.5.
