# Supplementary material for: Lysine-Triggered Polymeric Hydrogels with Self-Adhesion, Stretchability, and Supportive Properties
Source: Polymers (Basel). 2024 May 13;16(10):1388. doi: 10.3390/polym16101388 (PMC11125877; doi:10.3390/polym16101388)
Supplement: Supplementary file 1 [file polymers-16-01388-s001.zip › polymers-2915938-supp-done.pdf]

polymers

MDPI

Supplementary Materials

Lysine-Triggered Polymeric Hydrogels with Self-Adhesion, Stretchability, and Supportive Properties

Chieh-Yun Juan [1], You-Sheng Zhang [1], Jen-Kun Cheng [2,3,4], Yu-Hsu Chen [5,6,*], Hsin-Chieh Lin [7,8,*] and Mei-Yu Yeh [1,*]

[1] Department of Chemistry, Chung Yuan Christian University, No. 200, Zhongbei Rd., Zhongli Dist., Taoyuan City 320314, Taiwan; jieyunr@gmail.com (C.-Y.J.); brian20010418@gmail.com (Y.-S.Z.)
[2] Department of Medical Research, MacKay Memorial Hospital, Taipei 10449, Taiwan; jkcheng@usa.net
[3] Department of Anesthesiology, MacKay Memorial Hospital, Taipei 10449, Taiwan
[4] Department of Medicine, MacKay Medical College, New Taipei City 25245, Taiwan
[5] Department of Orthopedic Surgery, Taoyuan General Hospital, Ministry of Health and Welfare, Taoyuan 330215, Taiwan
[6] Department of Biology and Anatomy, National Defense Medical Center, Taipei 114201, Taiwan
[7] Department of Materials Science and Engineering, National Yang Ming Chiao Tung University, Hsinchu 300093, Taiwan
[8] Center for Intelligent Drug Systems and Smart Bio-Devices (IDS^{2}B), National Yang Ming Chiao Tung University, Hsinchu 30068, Taiwan
* Correspondence: magister.yuhsu@gmail.com (Y.-H.C.); hclin45@nycu.edu.tw (H.-C.L.); myyeh@cycu.edu.tw (M.-Y.Y.)

Table S1. Design of formulation.[a]

	AAM[b]	PAAM[b]	DF-PEG[b]	LysMA[b]	Note
/	15.00	0.50	2.00	-	Figure S1 (a)
/	20.00	0.50	2.00	-	Figure S1 (b)
/	22.50	0.50	2.00	-	Figure S1 (c)
/	25.00	0.50	2.00	-	Figure S1 (d)
/	27.50	0.50	2.00	-	Figure S1 (e)[c]
/	30.00	0.50	2.00	-	Figure S1 (f)
/	27.50	0.00	2.00	-	Figure S2 (a)
/	27.50	0.25	2.00	-	Figure S2 (b)
/	27.50	0.50	2.00	-	Figure S2 (c) [c]
/	27.50	0.75	2.00	-	Figure S2 (d)
/	27.50	0.50	0.00	-	Figure S3 (a)
/	27.50	0.50	2.00	-	Figure S3 (b) [c]
/	27.50	0.50	4.00	-	Figure S3 (c)
/	27.50	0.50	6.00	-	Figure S3 (d)
**pADL0**	27.50	0.50	2.00	0.00	Figure 1 (a) [c]
**pADL0.5**	27.50	0.50	2.00	0.50	Figure 1 (b)
**pADL1**	27.50	0.50	2.00	1.00	Figure 1 (c)
**pADL1.5**	27.50	0.50	2.00	1.50	Figure 1 (d)
**pADL2**	27.50	0.50	2.00	2.00	Figure 1 (e)
**pADL2.5**	27.50	0.50	2.00	2.50	Figure 1 (f)
**pADL3**	27.50	0.50	2.00	3.00	Figure 1 (g)

[a] Potassium persulfate (0.15 % w/v), 1.0 mL deionized water, acrylamide is AAM, polyacrylamide is PAAM, dialdehyde-functionalized poly(ethylene glycol) is DF-PEG, and methacrylated lysine is LysMA. [b]unit: % w/v; [c]The same hydrogel.

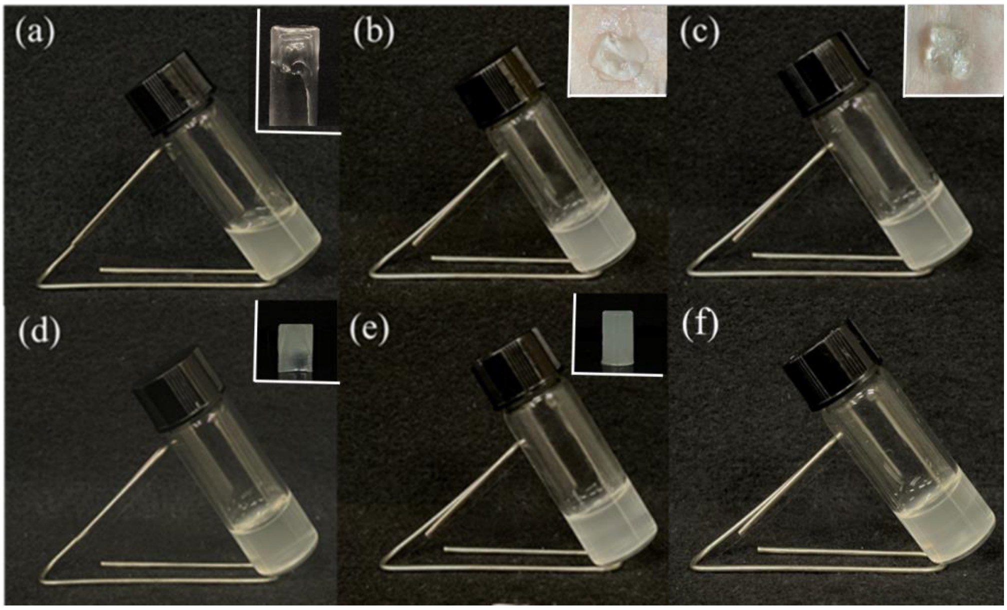

Figure S1. Optical images of hydrogels containing different % w/v of AAM. (a) 15.00 % w/v, (b) 20.00 % w/v, (c) 22.50 % w/v, (d) 25.00 % w/v, (e) 27.50 % w/v, and (f) 30.00 % w/v.

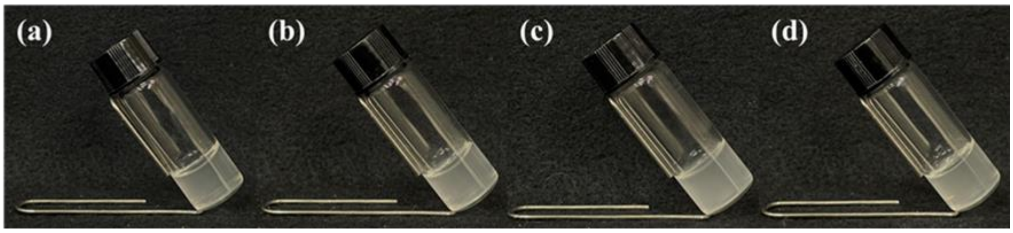

Figure S2. Optical images of hydrogels containing different % w/v of PAAM. (a) 0.00 % w/v, (b) 0.25 % w/v, (c) 0.50 % w/v, and (d) 0.75 % w/v.

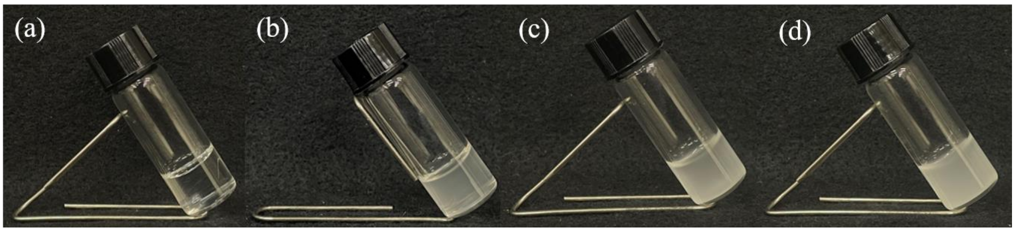

Figure S3. Optical images of hydrogels containing different % w/v of DF-PEG. (a) 0.00 % w/v, (b) 2.00 % w/v, (c) 4.00 % w/v, and (d) 6.00 % w/v.

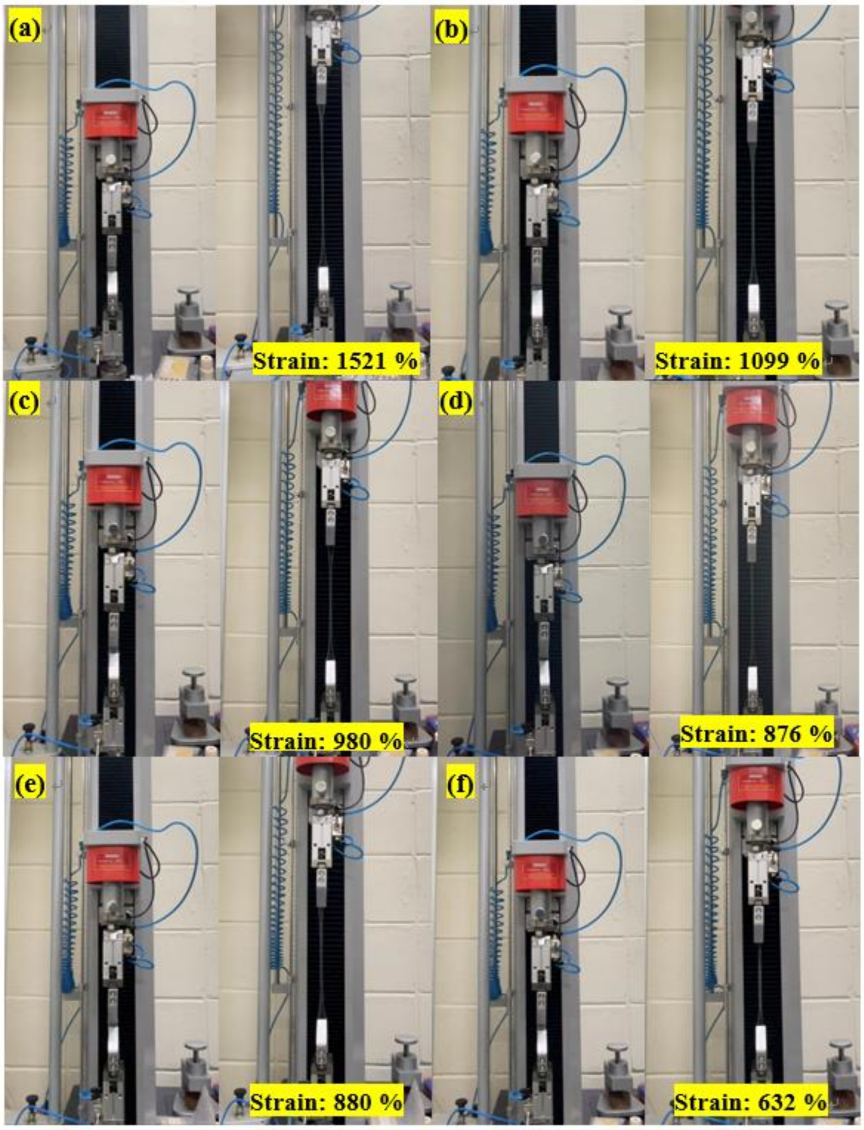

Figure S4. Comparison of tensile tests before and after stretching: (a) pADL0.5, (b) pADL1, (c) pADL1.5, (d) pADL2, (e) pADL2.5, and (f) pADL3. (Left: original length of 1 cm. Right: length after stretching).

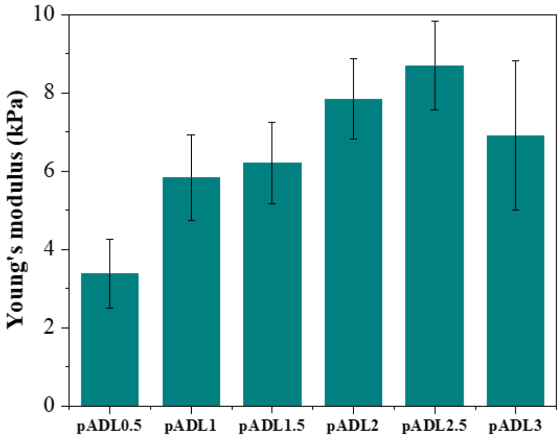

Figure S5. Young's modulus of pADL hydrogels.

Table S2. Mechanical properties of pADLx hydrogels.

	Tensile strength (kPa)	Elongation at break (%)	Toughness (kJ/m³)	Young's modulus (kPa)
**pADL0**	2.5	51	4.5	-
**pADL0.5**	5.9	1521	57.6	3.4
**pADL1**	11.3	1099	69.5	5.8
**pADL1.5**	10.8	980	61.1	6.2
**pADL2**	13.8	876	78.5	7.8
**pADL2.5**	18.9	880	98.8	8.7
**pADL3**	10.5	632	45.0	6.9

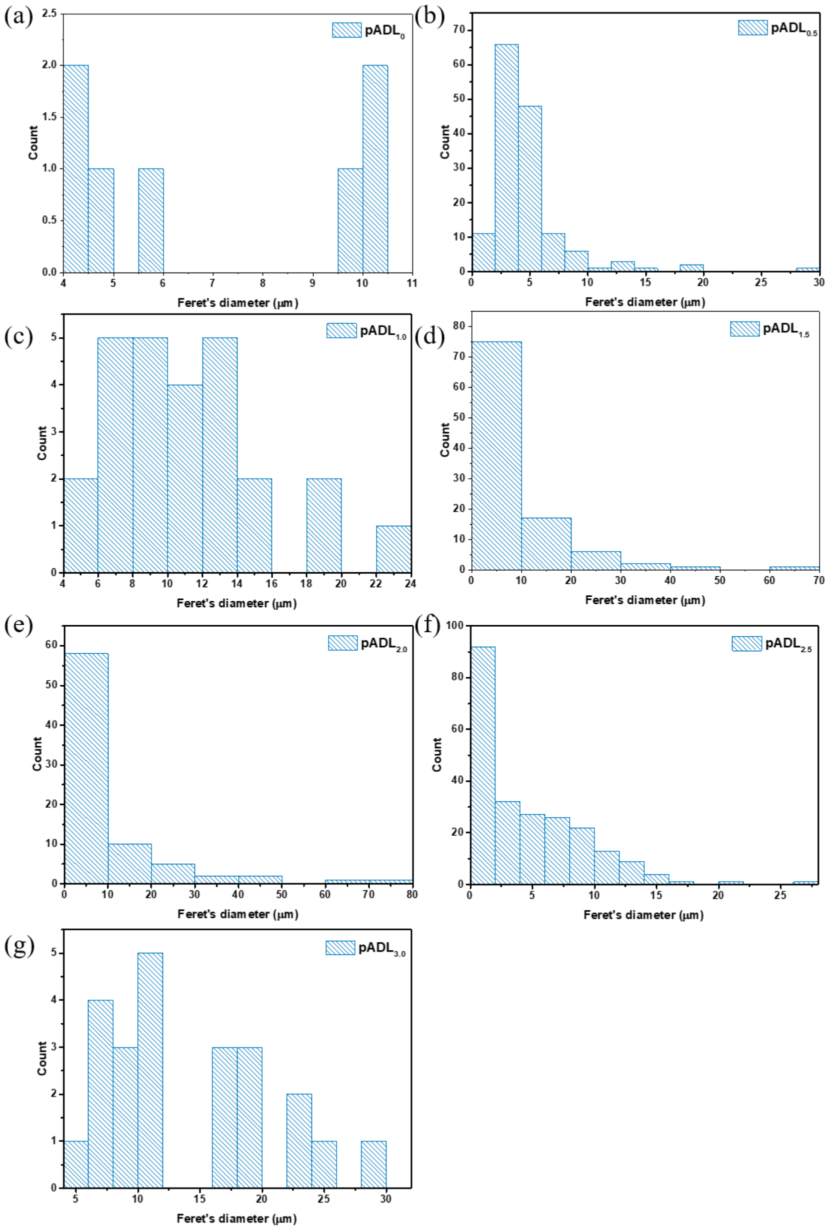

Figure S6. The porous diameter distributions of hydrogels of (a) pADL0, (b) pADL0.5, (c) pADL1, (d) pADL1.5, (e) pADL2, (f) pADL2.5, and (g) pADL3.

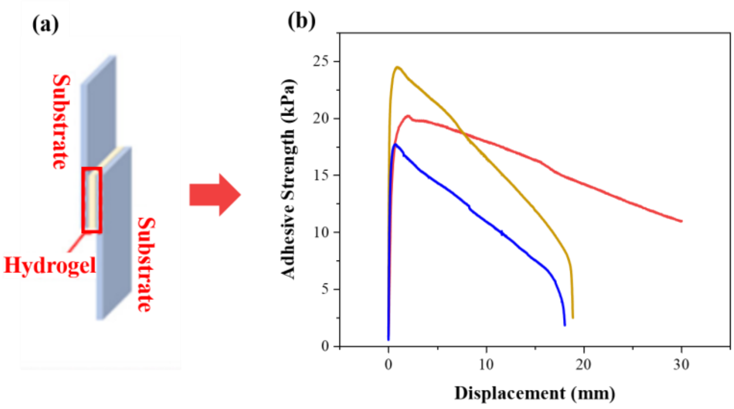

Figure S7. Lap-shear strength test of the pADL2.5 hydrogel on various substrates (red for glass, blue for skin, and brown for aluminum).

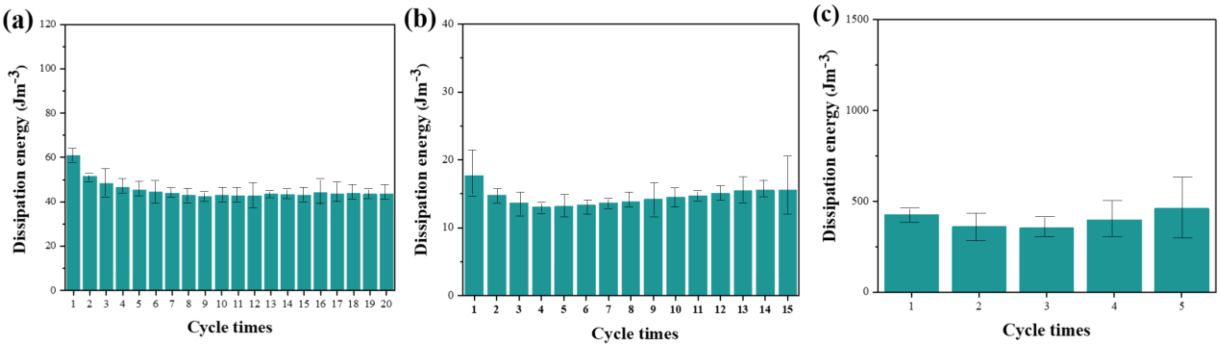

Figure S8. The dissipated energy of the pADL2.5 hydrogel: (a) 200% for 20 cycles, (b) 500% for 15 cycles, and (c) 800% for 5 cycles.

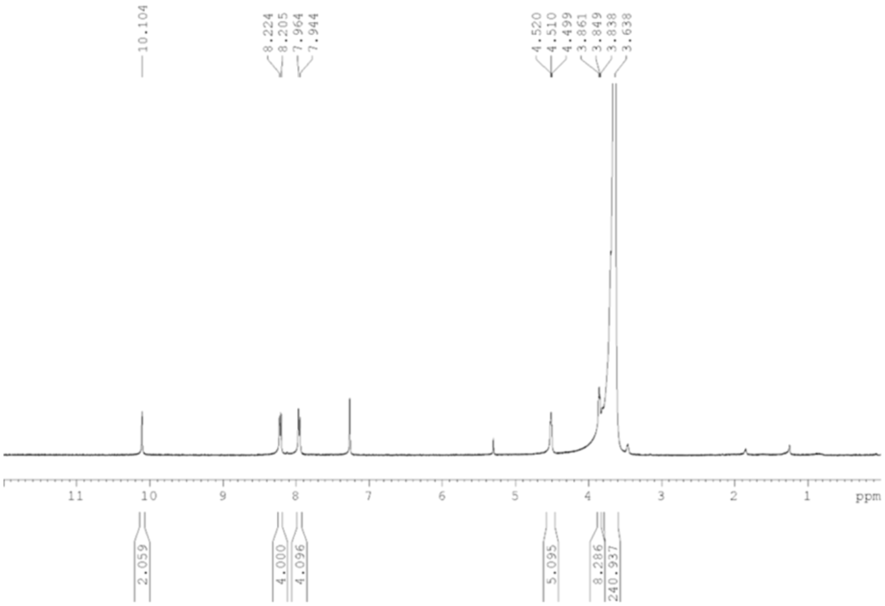

Figure S9. ^{1}H NMR spectrum of DF-PEG in $CDCl_3$.

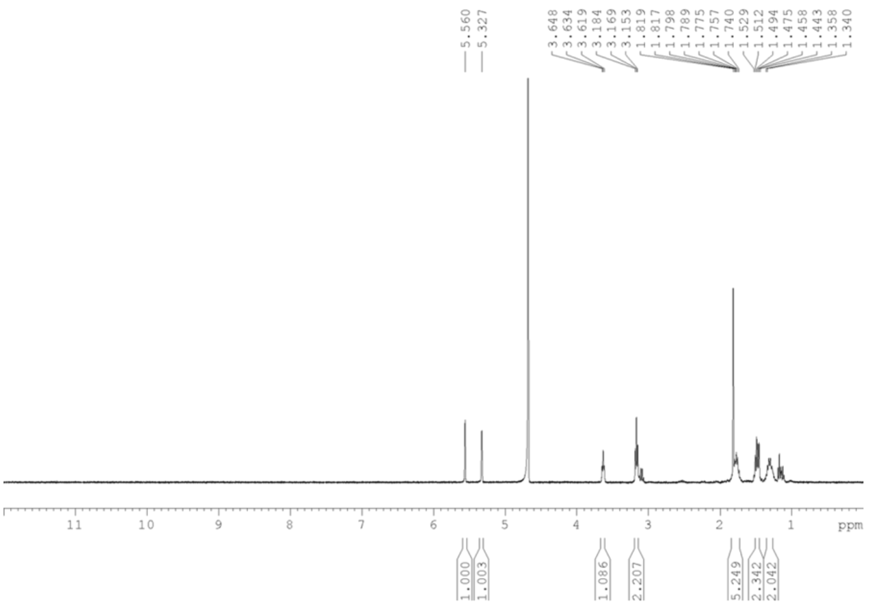

Figure S10. ^{1}H NMR spectrum of LysMA in D_2O.
